# Trends in Language Use During the COVID-19 Pandemic and Relationship Between Language Use and Mental Health: Text Analysis Based on Free Responses From a Longitudinal Study

**DOI:** 10.2196/40899

**Published:** 2023-03-01

**Authors:** Rachel Weger, Juan Antonio Lossio-Ventura, Margaret Rose-McCandlish, Jacob S Shaw, Stephen Sinclair, Francisco Pereira, Joyce Y Chung, Lauren Yvette Atlas

**Affiliations:** 1 National Center for Complementary and Integrative Health National Institutes of Health Bethesda, MD United States; 2 National Institute of Mental Health National Institutes of Health Bethesda, MD United States; 3 National Institute on Drug Abuse National Institutes of Health Baltimore, MD United States

**Keywords:** COVID-19, mental health, natural language processing, sentiment analysis, free response, qualitative, text analysis, mental illness, text, mental state, language, pandemic, age, education

## Abstract

**Background:**

The COVID-19 pandemic and its associated restrictions have been a major stressor that has exacerbated mental health worldwide. Qualitative data play a unique role in documenting mental states through both language features and content. Text analysis methods can provide insights into the associations between language use and mental health and reveal relevant themes that emerge organically in open-ended responses.

**Objective:**

The aim of this web-based longitudinal study on mental health during the early COVID-19 pandemic was to use text analysis methods to analyze free responses to the question, “Is there anything else you would like to tell us that might be important that we did not ask about?” Our goals were to determine whether individuals who responded to the item differed from nonresponders, to determine whether there were associations between language use and psychological status, and to characterize the content of responses and how responses changed over time.

**Methods:**

A total of 3655 individuals enrolled in the study were asked to complete self-reported measures of mental health and COVID-19 pandemic–related questions every 2 weeks for 6 months. Of these 3655 participants, 2497 (68.32%) provided at least 1 free response (9741 total responses). We used various text analysis methods to measure the links between language use and mental health and to characterize response themes over the first year of the pandemic.

**Results:**

Response likelihood was influenced by demographic factors and health status: those who were male, Asian, Black, or Hispanic were less likely to respond, and the odds of responding increased with age and education as well as with a history of physical health conditions. Although mental health treatment history did not influence the overall likelihood of responding, it was associated with more negative sentiment, negative word use, and higher use of first-person singular pronouns. Responses were dynamically influenced by psychological status such that distress and loneliness were positively associated with an individual’s likelihood to respond at a given time point and were associated with more negativity. Finally, the responses were negative in valence overall and exhibited fluctuations linked with external events. The responses covered a variety of topics, with the most common being mental health and emotion, social or physical distancing, and policy and government.

**Conclusions:**

Our results identify trends in language use during the first year of the pandemic and suggest that both the content of responses and overall sentiments are linked to mental health.

## Introduction

### Background

Times of crisis lead to increased psychological distress and mental health symptoms in the general population [1]. The literature from previous epidemics and emerging literature about the COVID-19 pandemic [2,3] provide an understanding of the mental health impacts of the COVID-19 pandemic. Increased psychological distress and mental illness is associated with a longer duration of quarantine [4,5], increased exposure to the virus or status as a health care worker [4,6,7], fear of infection of self or others [4,8], financial stress [4,9], preexisting mental illness [1,4,7], and social isolation [10,11]. It is critical to understand how this pandemic has affected mental health, document those effects, and prepare for future ones.

Language is one option for assessing mental health. Language and, more broadly, qualitative data can provide context for quantitative data and even point to new directions of research or uncover patterns that may not be found quantitatively. Language has been shown to predict states such as personality [12] and psychological constructs [13]. Research on mental health and language use has used machine learning to examine how language features correlate with or predict mental illness [14]. Other non–content-based metrics related to language have also been associated with mental illness, such as word count [15] and post counts on social media [16].

### Study Aims

**Figure 1:**
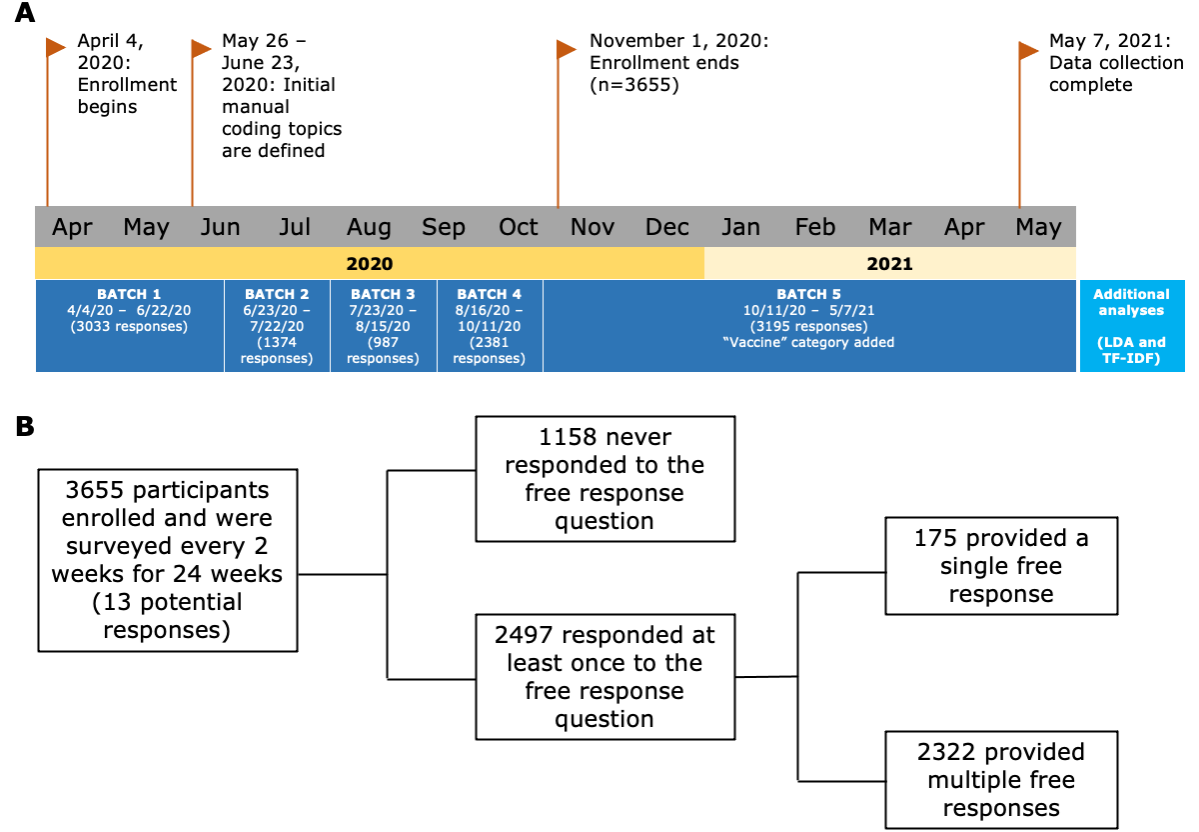
Schematic of study timeline. (A) Study and analysis timeline. Enrollment in the 6-month study proceeded from April 4, 2020, through November 1, 2020, and the final data point was collected on May 7, 2021. Manual coding analysis was conducted in 5 batches during data collection, whereas additional analyses (eg, latent Dirichlet allocation [LDA]) were conducted using the entire sample after data collection was complete. The batch numbers are listed with the number and dates of the responses they contained. (B) Participant free-response rate; 68% of participants provided at least one free response during the 6-month study, with 93% of these respondents providing multiple responses. TF-IDF: term frequency–inverse document frequency.

In this study, we examined data from a web-based mental health survey on COVID-19 stressors during the pandemic. The survey ended with an open-text free-response prompt, “Is there anything else you would like to tell us that might be important that we did not ask about?” (see Figure 1 for the study overview). Free-response questions have been shown to add context to and validate existing quantitative measures [17]. We used a variety of text analysis methods on these free responses to investigate the characteristics of our sample population, the content of responses, how responses changed over time, and how language use reflected the participants’ mental state.

To characterize the participants, we measured whether the demographics of the participants who responded to the free-response question differed from those who chose not to respond. On the basis of prior literature on responses to free-text comments in surveys, we predicted that, relative to those who did not respond, respondents would be more likely to be women, be older, have more years of education, and have a preexisting health condition [17-19]. In addition, we were interested in whether prior mental illness affected responses and language features. On the basis of prior work, we hypothesized that individuals with a history of mental health conditions would be more likely to respond and provide longer responses than those without mental health histories [15,16,20]. We also hypothesized that mental health history would be associated with more negative sentiment [15,16], greater use of negative emotional words [21-25], and more first-person singular pronouns (FPSPs) [16,20-30]. The rationale behind these selections is that increased FPSP use is associated with increased self-focus and increased negative valence and negative emotional words are associated with negatively biased thinking patterns [31,32]. These patterns of thought are associated with several mental disorders, including depression [33].

As for the content of responses, we first asked how the sentiment of responses varied over the course of the pandemic across all participants. On the basis of the literature from previous epidemics showing that distress increased with increased quarantine duration [4,5], we expected the sentiment to become more negative as social distancing and lockdown procedures remained in place. In addition, we expected emotional states to shape responses such that response likelihood and valence would be associated with fluctuations in self-reported loneliness, distress, and the presence of symptoms related to mental illness. Finally, we used various methods to categorize the responses.

## Methods

### Recruitment and Study Overview

A web-based, longitudinal study (NCT04339790) assessing the mental health impact of the COVID-19 pandemic was launched by investigators at the National Institute of Mental Health Intramural Research Program in early April 2020 (Figure 1). A convenience sample of adults aged ≥18 years was recruited via listserves, social media, word of mouth, flyers, and ClinicalTrials.gov (for more details, see the study by Chung et al [34]). After consenting on the web, participants completed self-report surveys upon enrollment and were then requested to respond to follow-up surveys every 2 weeks for 6 months. All survey data and responses were anonymized and associated with a unique ID.

### Ethics Approval

This study was approved by the Institutional Review Board of the National Institutes of Health (NIH; 20 M-N085).

### Questionnaires and Demographic Measures

At baseline, the participants completed various questionnaires assessing demographics, clinical history, and mental health symptoms (see the study by Chung et al [34] for a full list of study questionnaires). Then, they were invited to complete biweekly (ie, every 2 weeks) multiple-choice questionnaires for a 6-month period, including The Psychosocial Impact of COVID-19 Survey [35], which consisted of 45 multiple-choice questions that assess various attitudes, behaviors, and impacts surrounding the COVID-19 pandemic and a single free-response question (“Is there anything else you would like to tell us that might be important that we did not ask about?”). We analyzed responses to the free-response item and tested for associations with baseline demographics and clinical history questionnaires (see Multimedia Appendix 1 for details of classification of demographics and mental or physical health history [36-43]) as well as biweekly measures of loneliness, as measured by the University of California, Los Angeles 3-Item Loneliness Scale [44], and psychological distress, as measured by the Kessler-5 [45]. Participants could complete a maximum of 13 survey responses, one at each study time point. Of 2497 participants who provided free responses at any time point, 0.6% (n=15; range 2-6) of individuals provided duplicate responses across the study weeks. These individuals and their responses were included in the analyses because they covered stable concerns, such as employment, clinical conditions, physical health, and living situations.

### Language Analyses

#### Sentiment Analysis and Analysis of Language Features

Sentiment analysis algorithms process text and automatically calculate the emotionality or sentiment of that text. They may simply report whether the overall text is positive or negative or use a continuous scale that quantifies both valence and intensity. To determine the optimal algorithm for the free-response data, the responses were tokenized into sentences, preprocessed, and inputted into 8 commonly used sentiment analysis applications: Stanza [46], VADER [47], LIWC2015 [48], SentiStrength [49], TextBlob [50], NLPTown model [51], Pysentimiento [52], and TweetEval [53]. We also used singular value decomposition (SVD) and a majority vote measure, which combined the outputs of 8 applications into a continuous and categorical aggregate score. Of all 10 possible options for sentiment analysis (8 different tools and aggregation of their predictions using either SVD or a majority vote), TweetEval performed the best. It obtained a precision of 0.76, recall of 0.75, *F*_1_-score of 0.75, and accuracy of 0.80 and was therefore selected to measure sentiment. TweetEval is a roBERTa-based model [54] trained on approximately 60 million tweets. TweetEval represents the sentiment of the text on a scale of −1 to 1, with −1 being the most negative and 1 being the most positive.

We performed a formal evaluation by assigning a polarity category to 130 sentences drawn at random, which were manually labeled by a separate observer. This allowed us to compute *F* score, precision, and recall to compare the 8 polarity scores of the algorithms; the SVD score; and a majority vote of the 8 polarity scores. The TweetEval score outperformed the other options and was therefore selected for further analyses.

The TweetEval values were aggregated by response so that each response had a score that was the mean of the sentence-level TweetEval values. Then, those scores were aggregated by date so that each date had a mean TweetEval value. The 7-day rolling averages of TweetEval and the number of responses were computed using the *zoo* package and plotted by date [55].

In addition to sentiment, we focused on 3 additional language features of interest: word count (“WC” in LIWC2015 software), percentage of negative emotional words (“negemo”), and percentage of FPSPs (“i category”), which were calculated using the output from LIWC2015 [48].

#### Manual Content Analysis

We used manual content analysis to evaluate the responses in addition to automated algorithms. Two clinicians and 4 other members of the research team (SS, JYC, LYA, Molly Cosgrove, RW, and MR-M) created initial manual content analysis categories. One clinician and 5 other members of the research team (SS, LYA, Molly Cosgrove, RW, MR-M, and JSS) annotated 4 small practice batches and met after each to discuss ambiguities and refine categories and definitions. In total, 36 categories and definitions were agreed upon and sorted into 6 overarching themes: mental health, physical health, social factors, career and finances, society (including government, community, or both), and other. A complete list of the categories is presented in Multimedia Appendix 1. Free responses were divided among the 4 coders (SS, MR-M, RW, and JSS), and each response was reviewed and scored by 2 randomly selected coders. Each coder labeled the responses based on their content as belonging to ≥1 manual categories. The responses were annotated in 5 batches. For the fifth batch, with a date range from October 11, 2020, to May 5, 2021, LYA annotated instead of MR-M and the category “Vaccines” was added based on a consensus of the coders after noting changes in the themes of responses. Responses such as “No,” “NA,” and “Nothing to report” were not categorized by any coder and were classified as nonresponses that were removed from subsequent analyses. Clinicians (SS and JYC) reviewed responses marked as clinically significant to evaluate severity.

To assess agreement between coders, interrater reliability (IRR) was calculated using the *irrCAC* package to find both the Fleiss κ and Gwet AC1 statistic [56]. The 2 methods were chosen to complement each other because the κ statistic is very commonly used for IRR, whereas the Gwet AC1 statistic overcomes some of the κ statistic’s weakness with data with low variability [57,58]. For both measures, we evaluated agreement using the 1991 Altman interpretation of the κ statistic, in which <0.2 is poor, 0.2 to 0.4 is fair, 0.4 to 0.6 is moderate, 0.6 to 0.8 is good, and 0.8 to 1.0 is very good agreement [59].

#### Automated Topic Analysis

To supplement automated coding and manual scoring in predetermined categories, we used exploratory analyses to identify the topics that emerged in the responses over time. We focused on terms unique to each month. We used term frequency–inverse document frequency (TF-IDF), a technique that finds the words that appear the most frequently in 1 document (ie, all words for a given month) and the least frequently in the others (ie, all other months). Words were lemmatized using the *textstem* package [60], and TF-IDF was calculated using the *tidytext* package [61]. This analysis was performed independently from our manual content analysis to address topics that might have been omitted from our manual content analysis, for which categories were selected early in the pandemic and analysis proceeded in real time relative to data collection. The lemmas "coronavirus," "covid19," and "covid" were all classified as the lemma "covid," and the lemmas "vaccination," "vaccinate," and "vaccine," were all classified as "vaccine". 

Finally, we used natural language processing methods, such as topic modeling and multiword expression extraction, to explore people’s thoughts and concerns during the COVID-19 pandemic. Topic modeling automatically identifies clusters of words and themes from text data sets. One of the most popular methods is latent Dirichlet allocation (LDA), which seeks to classify text documents as a mixture of distinct topics [62] and has been widely used in automatic content analysis. The advantages of topic modeling are its high scalability and ability to infer topics or themes without being biased by users. However, a limitation is the potential lack of interpretability. This can occur because perplexity—a measure to evaluate the quality of topic modeling outputs and select model parameters—may be inversely correlated with human interpretability [63,64].

Therefore, the incorporation of additional evaluations or measures to validate the comprehensibility of topic-modeling outputs, such as human judgment [65,66], is necessary. In this study, we added human judgment in 3 steps to overcome the lack of interpretability. First, we used different values for the number of topics, calculated the performance for each iteration using perplexity, and compared the results to the manual number of themes found earlier by the annotators. Second, we calculated the agreement between the human and LDA topic assignments for over 100 sentences. We did this by randomly selecting 100 sentences and having 1 author sort them into the topics created by LDA. Then, the human- and LDA-selected topics were compared. Of the 100 sentences, 35 had complete agreement and 23 had weak agreement (the topic selections differed, but the author thought the LDA selection was reasonable or a closely related topic). There was no agreement among the remaining 42 sentences. Close inspection of sentences with disagreement revealed that in 14 cases, LDA-selected topics were based on keywords, but those words did not reflect the meaning of the sentence (eg, “DBT therapy has been big positive” was marked as topic 13 [relating to test results] by LDA likely because of the word “positive,” whereas the human coder rated it as topic 21 [relating to mental illness and medication]). Third, we intuitively evaluated the most representative keywords per topic (single-word terms extracted by LDA) by adding multiword terms to help represent topics better. Indeed, the output of topic modeling methods generally consists only of groupings of single-word terms. However, in natural languages, single-word terms are often part of multiword expressions and therefore do not provide complete context alone. Thus, an alternative to improve the identification of relevant topics is to incorporate multiword terms. These are expressions composed of ≥2 words with a grammatical structure and a specific meaning. Thus, we used LIDF-value [40], an information retrieval measure that extracts multiword terms. LIDF-value is based on several linguistic patterns also known as lexical categories such as nouns, adjectives, etc. Therefore, to automatically assess the content of the participants’ responses, our approach consisted of four basic steps: (1) preprocessing, (2) topic modeling with LDA, (3) multiword term extraction with LIDF-value, and (4) word cloud creation. Further details regarding the automated topic analysis can be found in Multimedia Appendix 1.

### Statistical Analyses

#### Comparing Respondents With Nonrespondents

A logistic regression was run comparing respondents and nonrespondents by gender, race, ethnicity, age, income, education, and preexisting mental health and medical conditions. Before running the logistic regression for age (the only continuous variable), the assumption of linearity between age and free-response response was tested. Participants’ ages were divided into quantiles, and logits were plotted by age category. The relationship was monotonic, therefore meeting the assumptions of logistic regression. R was used for all analyses, and *ggplot2* within the package *tidyverse* was used for all figures, except where noted [67,68].

#### Evaluating the Likelihood of Free Response and Sentiment as a Function of Psychological State

We used a multilevel logistic regression implemented using the function *glmer* in the R package *lme4* [69] to determine whether loneliness (measured using the University of California, Los Angeles 3-Item Loneliness Scale total score) and distress (measured using the Kessler-5 overall score) influenced the likelihood of an individual providing a free response at a given time point and whether the likelihood of responding changed over time.

We also used linear mixed models restricted to participants who provided multiple free responses (n=2322) to determine whether loneliness or psychological distress influenced the mean sentiment of an individual’s response at a given time point. Linear mixed models were implemented using the function *lmer* within the R package *lme4* [69]. We used a similar linear model to test whether free-response length (ie, number of words) varied over time and whether response length was related to loneliness or psychological distress. We also explored whether sentiment is associated with response length.

For each model, we included fixed effects of week (ie, time in the study relative to each participant’s time of enrollment), modeled psychological state both within- and between-subjects (ie, mean-centered within individuals and grand mean–centered across individuals), and included interactions between within- and between-subjects factors to test whether individual differences moderated the effects over time. Intercepts and slopes were treated as random in linear models, whereas logistic models included only random intercepts because of issues with model convergence. Because psychological predictors were correlated, we analyzed both combined models (reported in the main manuscript) and models that separately evaluated associations with loneliness and distress (reported in Multimedia Appendix 1).

#### Correlations Between Patient Clinical History and Language Features

We were interested in whether those with a history of mental health treatment, termed patients, used language features differently from controls. We focused on 4 language features of interest, each aggregated to be the mean by subject across all their responses over the course of the entire survey: sentiment, word count, percentage of negative emotional words, and percentage of FPSPs.

Two methods were used to determine mental health status. We determined whether an individual had a history of mental health concerns using a clinical history questionnaire. Patients were defined as individuals who reported prior mental health treatment including hospitalization, psychotropic medication, or treatment for drug or alcohol use. We used 2-sample *t* tests (2-tailed) to assess whether patients differed from controls (ie, individuals with no prior treatment for mental illness) for each language feature. Hedges *g* was calculated using the package *effsize* to show the effect size [70]. One participant did not complete the clinical history question at baseline and was therefore excluded from this analysis.

We also explored associations between language features and a continuous measure of each individual’s probability of being a patient, the patient probability score (PPS) [34]. PPS scores were trained on baseline questionnaire data from a subset of participants who were seen at the NIH before the pandemic and underwent a Structured Clinical Interview for DSM-5 (Diagnostic and Statistical Manual-5). Each participant who had not been seen at NIH was assigned a PPS value based on similarity to the patient or control group. For additional information and validation, refer to the study by Chung et al [34]. We used Spearman correlations to evaluate the associations between PPS and the 4 language features listed earlier. Seven participants were missing a PPS and were excluded from the analyses.

## Results

### Free-Response Sample

**Figure 2:**
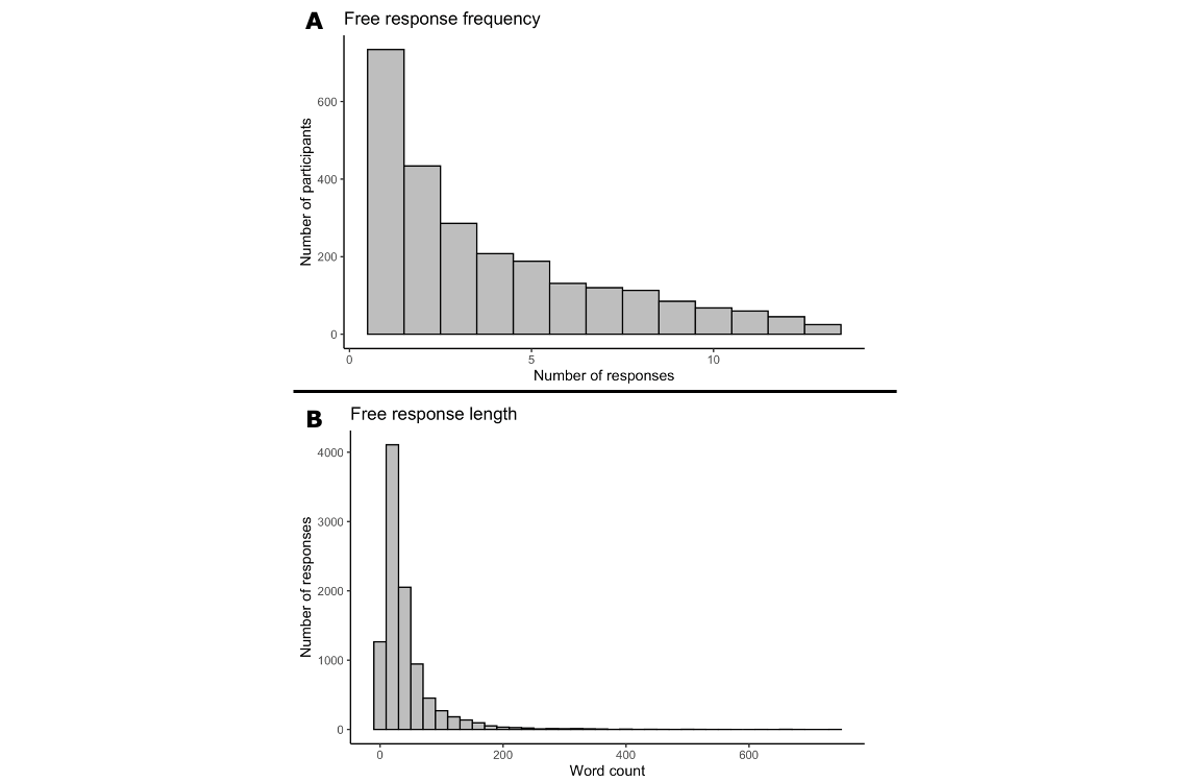
Distribution of free responses. (A) Response frequency. Histogram showing the number of participants who responded from 1 to 13 times to the free-response question. (B) Response length. Histogram showing the distribution of word count across all responses, which ranged from 1 to 744 words.

Of the 3655 participants enrolled in the study [34], 2497 participants responded at least once to the free-response item; these participants will be referred to as “respondents.” Figure 2 depicts the distribution of the respondents as a function of the number of times they provided free-response entries. The demographics of the total sample comparing respondents and nonrespondents (ie, participants who never provided free-response entries) are reported in Table 1. There was a total of 9738 free-response item responses.

**Table 1:** Comparison of respondents and nonrespondents^a^.

			Respondent, n (%)		Nonrespondent, n (%)		Responding, OR^b^ (95% CI)
**Gender**							
	Female	2065 (56.4)		866 (23.7)		1 (—^c^)	
	Male	346 (9.5)		255 (7)		0.57^d^ (0.48-0.68)	
	Nonbinary	58 (1.6)		21 (0.6)		1.16 (0.71-1.96)	
	Unknown	28 (0.8)		16 (0.4)		—	
**Ethnicity**							
	Hispanic or Latino	124 (3.4)		79 (2.2)		0.71^e^ (0.53-0.95)	
	Not Hispanic or Latino	2271 (62.1)		1026 (28.1)		1 (—)	
	Unknown	102 (2.8)		53 (1.5)		—	
**Race**							
	American Indian or Alaska Native	7 (0.2)		4 (0.1)		—	
	Asian	43 (1.2)		47 (1.3)		0.40^d^ (0.26-0.62)	
	Black or African American	62 (1.7)		44 (1.2)		0.62^e^ (0.42-0.93)	
	White or Caucasian	2256 (61.7)		997 (27.3)		1 (—)	
	Hawaiian or Pacific Islander	0 (0)		0 (0)		—	
	Multiple races	92 (2.5)		48 (1.3)		0.85 (0.60-1.22)	
	Unknown	37 (1)		18 (0.5)		—	
**Age (years)**							
	Values, mean (SD)	48.0 (14.9)		43.7 (14.4)		1.02^d^ (1.02-1.03)	
**Income (US $)**							
	<35,000	340 (9.3)		165 (4.5)		1 (—)	
	35,001-75,000	629 (17.2)		294 (8)		1.04 (0.82-1.31)	
	75,001-100,000	395 (10.8)		166 (4.5)		1.15 (0.89-1.50)	
	100,001-150,000	505 (16.4)		243 (6.6)		1.01 (0.79-1.28)	
	≥150,000	598 (16.4)		274 (7.5)		1.06 (0.84-1.34)	
	Unknown	30 (0.8)		16 (0.4)		—	
**Education**							
	Less than high school	4 (0.1)		6 (0.2)		—	
	High school graduate or above	52 (1.4)		41 (1.1)		1 (—)	
	Some college or above	179 (4.9)		130 (3.6)		1.09 (0.67-1.73)	
	Associate degree or above	111 (3)		71 (1.9)		1.23 (0.74-2.04)	
	Bachelor’s degree or above	788 (21.6)		366 (10)		1.70^e^ (1.10-2.60)	
	Advanced or professional degree	1355 (37.1)		540 (14.8)		1.98^f^ (1.29-3.01)	
	Unknown	8 (0.2)		4 (0.1)		—	
**Mental health status**							
	Mental health history	1384 (37.9)		609 (16.7)		1 (—)	
	No mental health history	1113 (30.5)		548 (15)		0.89 (0.78-1.03)	
	Unknown	0 (0)		1 (0)		—	
**Physical health status**							
	Has medical illness	1369 (37.5)		522 (14.3)		1 (—)	
	Does not have medical illness	1128 (30.9)		635 (17.4)		0.68^d^ (0.59-0.78)	
	Unknown	0 (0)		1 (0)		—	

^a^This table compares the demographics and clinical history of participants who responded at least once to the free-response question (“respondents”) and those who did not (“nonrespondents”). The odds ratio of responding for each group compared with the reference group is shown in the third column. The reference groups were denoted by those with an odds ratio of 1.

^b^OR: odds ratio.

^c^Groups with too small a sample size and those whose demographics were unknown were not included in the logistic regression.

^d^Mean values <0.001.

^e^Mean values between 0.05 and 0.01.

^f^Mean values between 0.01 and 0.001.

### Comparing Respondents With Nonrespondents

Logistic regressions indicated that the likelihood of responding was influenced by several demographic factors, including gender, race, ethnicity, education, and age, as reported in Table 1. For example, the odds of male participants responding compared with female participants were 43% lower (*P*<.001), and the odds of Asian and Black participants responding compared with White participants were 60% (*P*<.001) and 38% (*P*=.02) lower, respectively. Although education influenced the likelihood of responding such that the odds of participants with bachelor’s or advanced degrees responding compared with participants who were high school graduates were 70% (*P*=.02) and 98% (*P*=.002) higher, respectively, we did not observe any influence of income. For additional demographic factors, please refer to Table 1.

Interestingly, there was no impact of mental health history on an individual’s likelihood of providing free responses (Table 1). However, physical health history did influence an individual’s likelihood of providing free responses, and the odds of participants without physical health conditions responding compared with those with these conditions was 32% lower (*P*<.001).

### Impact of Psychological State on Likelihood of Providing a Free Response

We used multilevel models to evaluate the likelihood of an individual providing a response on a given week as a function of time and psychological state. All models revealed that an individual’s likelihood of providing a free-response decreased over time (Table 2), although the effects were quite small based on odds ratios. Individuals were more likely to respond to the free-response item when feeling more distressed, as measured by the Kessler-5, and individuals with higher average distress were more likely to respond. Interestingly, we observed an interaction between within-subjects distress and between-subjects distress such that the effect of distress on the likelihood of responding for a given week was strongest for individuals with low average distress, perhaps because individuals with high average distress responded consistently over time. There was no effect of loneliness on the likelihood of responding when it was included in the same model as the distress measure; however, the fixed effects of loneliness and distress were correlated across individuals (*r*=0.633), and we therefore computed separate models for each predictor (Multimedia Appendix 1). Modeling distress alone confirmed the findings from the model that included all factors with similar coefficients. When loneliness was included in a separate model, we found that individuals were more likely to respond when they reported higher loneliness (B=0.05; *P*=.004) and that individuals who reported being more lonely on average were more likely to respond (B=0.09; *P*<.001).

**Table 2:** Multilevel logistic model examining association among distress, loneliness, and likelihood of response^a^.

Predictor	OR^b^ (95% CI)	*P* value
(Intercept)	0.37 (0.34-0.40)	<.001
Week	0.98 (0.98-0.98)	<.001
Distress	1.13 (1.11-1.15)	<.001
Mean distress	1.03 (1.01-1.06)	.005
Loneliness	0.98 (0.95-1.02)	.35
Mean loneliness	1.05 (0.99-1.10)	.10
Distress × mean distress	0.99 (0.98-0.99)	<.001
Loneliness × mean loneliness	0.98 (0.96-1.01)	.18

^a^This table presents the results of a multilevel logistic model examining the association between the likelihood of response on a given week and self-reported distress (measured using the Kessler-5) and loneliness (measured using the 3-item Loneliness Scale). Distress and loneliness were modeled both within (ie, dynamic fluctuations across intervals) and between participants (ie, mean distress and mean loneliness). There were 26,073 observations across 3163 individuals, with intraclass correlation coefficient=0.49, marginal *R*^2^=0.017, conditional *R*^2^=0.495, random error variance (*σ*^2^)=3.29, and variance of random intercepts (*τ*_00__SUBJECT_NUMBER_)=3.11. The results of the models that separately analyzed distress and loneliness are presented in Multimedia Appendix 1.

^b^OR: odds ratio.

### Sentiment During the Study Period

**Figure 3:**
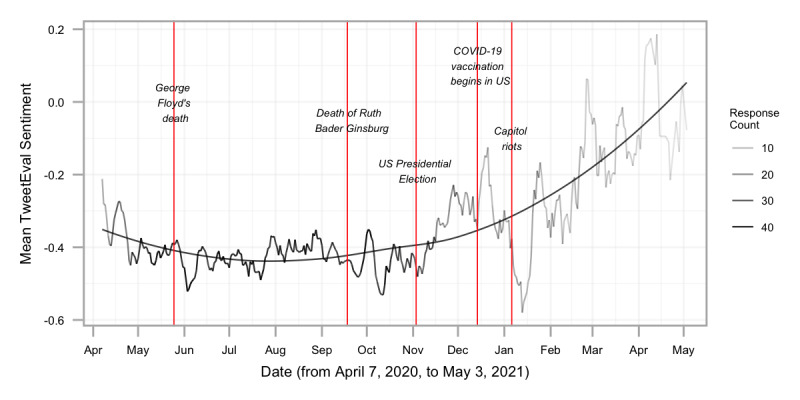
Sentiment over time: this figure plots the 7-day rolling average of sentiment by day from April 7, 2020, to May 3, 2021 (responses from before April 7, 2020, or after May 3, 2021, are omitted due to the 7-day rolling average). The opacity of the line represents the 7-day rolling average of response count. TweetEval Sentiment below 0 is considered negative. Red vertical bars mark the dates of major national events in the United States, which emerged in free-response comments based on term frequency–inverse document frequency.

The results of sentiment analysis are shown in Figure 3. As the TweetEval scores range from −1 to 1, it is clear from the figure that the average sentiment of free responses remained negative for the entire study period. We observed a gradual upward tendency in sentiment starting in November, which coincides with announcements about the Pfizer vaccine (Figure 3). However, our sample size and proportion of responses were reduced at this time, and we did not run statistical analyses on the influence of time on sentiment; therefore, we do not make strong inferences about these overall patterns based on group averages.

Important events throughout the pandemic that may have affected groupwide sentiment are marked in Figure 3. These events were selected based on the keywords seen in the TF-IDF analysis (see analysis below in Themes of Free Responses Across Time). The selected events were important events in the United States, given that most of the study participants came from the United States, with all 50 states represented; of free-response respondents, 2474 were based in the United States and 23 were international. The 5 events chosen were the death of George Floyd (May 25, 2020), the death of Ruth Bader Ginsburg (September 18, 2020), the 2020 US Presidential Election (November 3, 2020), the beginning of COVID-19 vaccination in the United States (December 14, 2020 [71]), and the US Capitol attack (January 6, 2021). As depicted in Figure 3, these events were followed by steep changes in the average sentiment of responses, as measured by TweetEval.

### Association Between Psychological State and Sentiment of Responses

**Figure 4:**
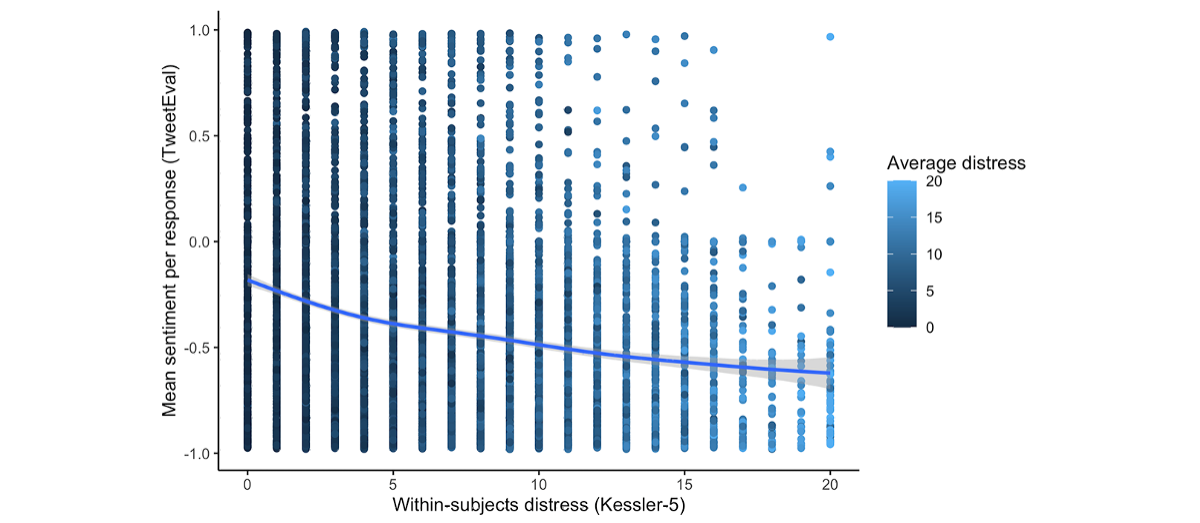
Association between distress (Kessler-5) and sentiment (TweetEval score) in free responses. Scatterplot illustrating association between biweekly measures of distress (as measured by Kessler-5) and mean sentiment of free responses as measured by TweetEval. Linear mixed models indicate that distress is negatively associated with sentiment within individuals, and that individuals with higher mean distress (visualized in lighter blue) use more negative language on average.

We used multilevel models to evaluate the dynamic association between self-reported psychological states and response sentiment, as measured by the mean TweetEval score per response. A model that combined distress and loneliness (Table 3) indicated that sentiment was negative on average, based on the intercept, and that sentiment increased over time within individuals, which is consistent with the overall average depicted in Figure 3. Responses were more negative at time points when individuals reported greater distress (Figure 4) or loneliness. We also observed that individuals with higher mean distress had more negative sentiment on average (Figure 4) and that there was a substantial interaction between within-subjects distress and between-subjects distress, such that the effect of distress on sentiment was strongest for those with low average distress scores. Between-subjects variations in loneliness did not influence sentiment when loneliness was included in the same model as distress; however, when loneliness and distress were modeled separately, we observed substantial associations with each measure, both within and between participants (Multimedia Appendix 1).

**Table 3:** Linear mixed model examining association among distress, loneliness, and likelihood of response^a^.

Predictors	Estimates (95% CI)	*P* value
(Intercept)	−0.385 (−0.396 to −0.373)	<.001
Week	0.002 (0.001 to 0.004)	<.001
Distress	−0.038 (−0.043 to −0.033)	<.001
Mean distress	−0.021 (−0.024 to −0.017)	<.001
Loneliness	−0.019 (−0.029 to −0.010)	<.001
Mean loneliness	0.002 (−0.007 to 0.010)	.68
Distress × mean distress	0.002 (0.001 to 0.004)	<.001
Loneliness × mean loneliness	−0.002 (−0.009 to 0.004)	.49

^a^This table presents the results of a linear mixed model examining the associations between negative sentiment, self-reported distress, and loneliness (see the *Methods* section). There were 9253 observations across 2314 individuals, with intraclass correlation coefficient=0.14, marginal *R*^2^=0.064, conditional *R*^2^=0.199, random error variance (*σ*^2^)=0.17, variance of random intercepts (*τ*_00__SUBJECT_NUMBER_)=0.03, and variance of random slopes=0. The results from the models that separately analyzed distress and loneliness are presented in Multimedia Appendix 1.

### Association Between Psychological State and Response Length

We tested whether response length varied as a function of loneliness, distress, and time (Table 4). Response length ranged from 1 word to a maximum of 744 words (mean 41.63, SD 49.05; median 27; Figure 2). Linear mixed models indicated that response length decreased slightly over the course of an individual’s participation, such that each biweekly interval was 0.5 words shorter on average. Response length was positively associated with distress (Table 4), such that an increase of 1 unit of distress on a given week was associated with an additional 0.8 words, and individuals who report higher distress provided responses that were 0.8 words longer on average. There was no effect of loneliness on response length when distress and loneliness were included in the same model, but separate analyses indicated that responses were longer in lonely individuals than in nonlonely individuals (*P*<.001), such that an increase of one unit in average loneliness was associated with 1.3 more words on average (Multimedia Appendix 1).

**Table 4:** Linear mixed model examining association among distress, loneliness, and response length^a^.

Predictors	Estimates (95% CI)	*P* value
(Intercept)	36.726 (35.294 to 38.157)	<.001
Week	−0.249 (−0.358 to −0.140)	<.001
Distress	0.811 (0.361 to 1.261)	<.001
Mean distress	0.827 (0.371 to 1.282)	<.001
Loneliness	−0.081 (−0.988 to 0.826)	.86
Mean loneliness	0.166 (−0.867 to 1.200)	.75
Distress × mean distress	−0.044 (−0.162 to 0.073)	.46
Loneliness × mean loneliness	−0.235 (−0.851 to 0.381)	.46

^a^This table presents the results of a linear mixed model examining associations between word count, self-reported distress, and loneliness (see the *Methods* section). There were 9272 observations across 2314 individuals, with intraclass correlation coefficient=0.33, marginal *R*^2^=0.009, conditional *R*^2^=0.340, random error variance (*σ*^2^)=1406.63, variance of random intercepts (*τ*_00__SUBJECT_NUMBER_)=705.36, variance of random slopes for distress=1.96, and variance random slopes for loneliness=17.25. The results from the models that separately analyzed distress and loneliness are presented in Multimedia Appendix 1.

### Associations Between Mental Health History and Language Features

As reported in Table 5, individuals who reported prior mental health treatment had more negative sentiments (as measured by TweetEval), wrote longer responses, used more negative emotional words, and had higher frequencies of FPSP use. The effect sizes and *P* values are presented in Table 5. We also observed small but substantial associations with language features when we used our continuous PPS: PPS was associated with more negative sentiment (*r*=−0.12; *P*<.001), higher word counts (*r*=0.09; *P*<.001), more negative emotional words (*r*=0.04; *P*<.05), and higher FPSP use (*r*=0.13; *P*<.001).

**Table 5:** Relationship between mental health history and language use^a^.

	Sentiment (TweetEval)	Word count	Negative emotional words (%)	First-person singular pronoun (%)
Mental health history, mean (SD)	−0.0044 (0.0034)	37.1 (33.8)	5.53 (6.15)	7.46 (4.77)
No mental health history, mean (SD)	−0.0038 (0.0036)	34.2 (30.2)	4.77 (4.42)	6.56 (4.46)
*P* value	<.001	.03	<.01	<.001
Hedges *g* effect size	−0.15	0.09	0.14	0.19

^a^A 2-sample *t* test (2-tailed) was run to compare the use of 4 language features by mental health history, as determined by mental health or drug or alcohol treatment or mental health hospitalization. The language features selected were the same as those used in the Spearman correlation analysis.

### Themes of Free Responses Across Time

The results of the manual coding are reported in Table 6. The most frequently annotated categories were mental health or emotion (5159/9738, 53% of responses), social or physical distance (2475/9738, 25% of responses), and policy or government (1938/9738, 20% of responses). The Fleiss κ coefficient for IRR for all responses was 0.73 (95% CI 0.72-0.73), which is characterized as “good” agreement between raters [59]. The Gwet AC1 statistic coefficient for IRR for all responses was 0.96 (95% CI 0.96-0.96) or “very good” agreement [59]. Agreement for individual categories is presented in Multimedia Appendix 1. Only 2 categories (“non–health-related concern for the immediate circle” and “clarification of survey response”) were characterized as “fair” by the Fleiss κ statistic; all others ranged from moderate to very good agreement.

**Table 6:** Manual coding of free-response topics^a^.

Theme and category		Example response	Coding count (N=9738), n (%)
**Mental health**			
	General negative mental health (ie, negative emotion or cognitive symptom)	“I have experienced a lot of physical symptoms of stress/anxiety, including fatigue and pain.”	5159 (53)
	Clinically significant (eg, mention of diagnosis, treatment, suicidality, or domestic violence)	“I have been on Prozac for 3 weeks now”	1837 (9)
	Mood disorder	“I’m experiencing some depression, but I’m not having suicidal thoughts. I have a hard time thinking about a future that is different than it is now.”	475 (5)
	Anxiety disorder	“I took wellbutrin a few years ago, started a new RX this week for anxiety over Covid”	309 (3)
	Other psychiatric diagnosis	“PTSD^b^ symptoms re-activated by feeling trapped, uncertainty, amount of unknowns, untrustworthy authority figure”	275 (3)
	Suicidality	“had to call suicide prevention hotline due to crisis”	130 (1)
**Physical health**			
	Non–COVID-19–related physical health	“Symptoms listed above due to chronic asthma/allergies”	1444 (15)
	Change in health behaviors, activities, or hobbies	“I am eating more and gaining weight”	798 (8)
	Suspected or confirmed COVID-19 illness or self-test	“I did antibody testing with a home-kit because I was worried about my symptoms”	486 (5)
	Sleep	“My sleep schedule has been completely thrown off.”	366 (4)
	Deferred medical care	“Close friend diagnosed with cancer and treatment was delayed due to pandemic so it spread faster than expected.”	159 (2)
	Drugs and alcohol	“Watching way too much TV; and smoking a heck of a lot more than I used to.”	154 (2)
	COVID-19–related risk factors in self	“Constant worry because I have asthma/COPD^c^”	149 (2)
	Pregnancy	“I am pregnant with my first child and am very nervous about contracting COVID or my partner contracting COVID.”	42 (1)
**Social factors**			
	Experience with social or physical distance and masks	“extended family has different beliefs about social distancing which increase stress”	2475 (25)
	Health condition or health-related concern about immediate circle	“Most of my stress is related to a sick family member (not Covid)”	1697 (17)
	Providing care for dependents	“It is increasingly challenging to work full-time and parent children who are attending school at home.”	570 (6)
	Strained relationships	“My relationship with my spouse has been more rocky. It’s been a lot to rely on one introverted person for my extroverted needs.”	547 (6)
	Non–health-related concern for immediate circle	“I’m anxious about the fears and anxieties of my closest friend. He’s not handling the virus threat well at all.”	513 (5)
	Mention of non–COVID-19–related death	“Mother passed away from pancreatic cancer”	393 (4)
	Loneliness or isolation	“I am beginning to notice the lack of and to miss physical presence and physical contact with people – besides my partner.”	308 (3)
	Positive relationships	“I live in a beautiful place with my wife, who is the love of my life.”	172 (2)
	Mention of COVID-19–related death	“My sister in law died from COVID-19 after 23 days in ICU^d^. Buried her last Saturday.”	95 (1)
**Career and finances**			
	Other work-related issues	“It has been more difficult to focus on work while working from home.”	1545 (16)
	School-related changes (student or teacher)	“Back to work as a teacher. Very stressful because of fear of getting sick and because of changes in responsibilities and increased work load.”	716 (7)
	Experience or concern about reduction or loss in work or unemployment	“The biggest stressor is waiting to find out if I’m going to be laid off and worrying for my kids’ futures in terms of getting a job.”	491 (5)
	Essential worker or in health care	“I am tested weekly for COVID due to my work in assisted living facilities”	357 (4)
	Personal finances	“Approximate 85% of my COVID stress is related to financial uncertainty.”	317 (3)
**Society or government or community**			
	Policy or government	“My current distress and hopelessness is largely driven by the murder of George Floyd.”	1938 (20)
	Reopening or return to work and interactions with community	“My state is starting to reopen, but I think it’s too soon.”	1047 (11)
	Effects of pandemic on the economy or society	“I feel so sad and scared for the world right now.”	446 (5)
**Other**			
	Positive aspects	“I feel extremely lucky to be healthy, housed and have no financial or relationship worries.”	1291 (13)
	Survey feedback	“I think inquiring about dietary intake and weight change would be interesting.”	488 (5)
	Other	“I am a veterinarian”	485 (5)
	Vaccine	“I’ve received my first dose of the COVID vaccine, and a number of my family members are now fully vaccinated. That’s given me some hope.”	416 (4)
	Clarification of survey response	“I checked the box for using marijuana, but I only use CBD^e^ for sleep and back pain. CBD was not an option.”	362 (4)
	Minimal change to lifestyle	“I am a loner by nature so self-isolating is not a problem for me.”	55 (1)

^a^This table presents the frequency and percentage of responses manually annotated as a function of category, as well as example responses. For the original category names, definitions, and interrater reliability by category, see Multimedia Appendix 1.

^b^PTSD: posttraumatic stress disorder.

^c^COPD: chronic obstructive pulmonary disease.

^d^ICU: intensive care unit.

^e^CBD: cannabidiol.

**Figure 5:**
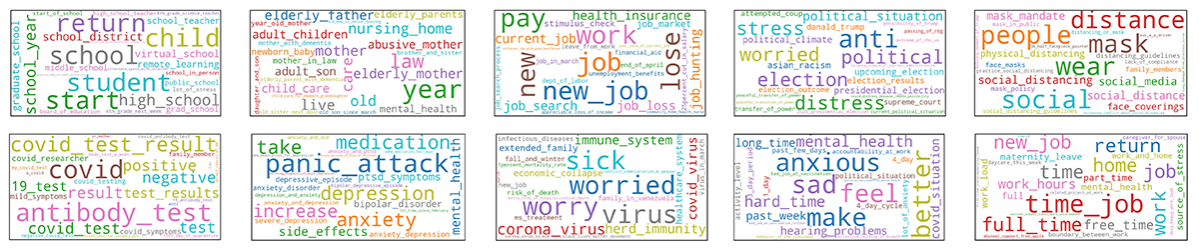
Most frequent topics based on topic modeling: the top 10 most frequent topics, depicted as word clouds. The word clouds contain the top 5 single-word terms per topic as identified by latent Dirichlet allocation and the top 20 multiword terms as identified by Linguistic patterns, inverse document frequency, and C-value information.

We also used TF-IDF to identify unique topics that emerged over time during the first year of the pandemic and therefore would not have been captured by our free-response categories. Table 7 presents the lemmas with the highest TF-IDF scores for each month. This analysis confirms the link between free responses and national and international events, such as the initial “lockdown” during the months of April and May 2020; responses to the killing of George Floyd in June 2020; wildfires and the death of Ruth Bader Ginsberg in September 2020; the lead up to the November 3, 2020, election; and the widespread availability of vaccines starting in February 2021. These events are visualized in Figure 3 to highlight the associations between events and the overall sentiment of the language. We also present word clouds of language use in Figure 5.

**Table 7:** Results of term frequency–inverse document frequency (TF-IDF) per month^a^.

Month and lemma		Term frequency, n	TF-IDF
**April 2020**			
	Time	146	0.0010
	Home	128	0.0009
	Distance	58	0.0008
	Guilt	12	0.0008
	Worry	108	0.0007
**May 2020**			
	Distance	107	0.0008
	Home	204	0.0008
	Time	188	0.0007
	Care	90	0.0007
	Patient	29	0.0007
**June 2020**			
	George	60	0.0045
	Floyd	50	0.0038
	Protest	159	0.0026
	Brutality	30	0.0023
	Riot	48	0.0020
**July 2020**			
	Wear	115	0.0008
	Care	105	0.0007
	People	191	0.0006
	Time	191	0.0006
	Distance	88	0.0006
**August 2020**			
	Time	187	0.0007
	Distance	88	0.0007
	Wear	84	0.0007
	College	36	0.0006
	Min	6	0.0006
**September 2020**			
	Wildfire	37	0.0017
	Rbg	20	0.0017
	Fire	47	0.0015
	Election	75	0.0014
	Ruth	9	0.0013
**October 2020**			
	Election	124	0.0025
	Supreme	9	0.0011
	Political	50	0.0007
	Time	162	0.0007
	Upcoming	25	0.0007
**November 2020**			
	Election	301	0.0102
	Thanksgiving	36	0.0025
	Presidential	30	0.0021
	Biden	29	0.0016
	Holiday	29	0.0016
**December 2020**			
	Christmas	49	0.0057
	Holiday	45	0.0042
	Thanksgiving	30	0.0035
	Election	22	0.0012
	Test	70	0.0009
**January 2021**			
	Capitol	60	0.0215
	Insurrection	32	0.0115
	Inauguration	18	0.0065
	Coup	13	0.0025
	Riot	13	0.0020
**February 2021**			
	Variant	15	0.0062
	Moderna	8	0.0027
	Shoot	23	0.0026
	21	7	0.0008
	Time	31	0.0008
**March 2021**			
	Shot	9	0.0039
	Moderna	8	0.0035
	Shoot	20	0.0029
	Pfizer	7	0.0021
	People	31	0.0010

^a^The top 5 lemmas with the highest TF-IDF scores per month are listed.

### Topic Model

The word clouds representing the 10 most frequent topics are shown in Figure 5. Although there is a substantial overlap with the most common topics found by manual coding (eg, topics relating to mental health, government, and social distancing), there are also several topics that build upon the results of manual coding by providing further context or identifying new topics (eg, various topics relating to work and family). All word clouds can be found in Multimedia Appendix 1.

## Discussion

### Principal Findings

We examined the use of a free-response question among individuals enrolled in a study on mental health during the COVID-19 pandemic, both in terms of the characteristics of its respondents and the content of their responses. Participants who responded to the free-response question were less likely to be male, Asian, Black, or Hispanic. They were also more likely to respond the older they were, the more education they had, and when they had physical health conditions. We found that, although participants with a mental health treatment history were not more likely to respond than those without prior histories, they had more negative sentiments, used more FPSPs and more negative emotional words, and provided longer responses. Participants responded more negatively and provided longer responses when they felt more distressed. Loneliness also predicted negative sentiment and an individual’s likelihood of responding at a given time point. The overall sentiment of the sample responses remained stable and constantly negative over the period of most responses from May 2020 to November 2020. However, there were marked fluctuations that seem to have been related to external events, which were also mentioned in the free responses based on our analyses of unique topics per month. Finally, most of the responses addressed negative emotional and mental health impacts, experiences with social distancing, and political and national events. Here, we discuss these findings and their implications for mental health during the COVID-19 pandemic, as well as future studies on text analysis in mental illness.

### Free Response Likelihood Varied Based on Demographic Factors

Similar to many other survey-based studies assessing the impact of the COVID-19 pandemic on physical and mental health and daily life [72-74], our overall study population was skewed, with respondents who were mainly White, educated, and female [34]. Interestingly, even within this skewed sample, those who responded to the free-response question were even more likely to be female, White, non-Hispanic, more educated, and older. Those who responded to the free-response question were also more likely to have a history of physical illness. These findings are consistent with previous studies of responses to free-response questions in patient satisfaction surveys [17-19]. Demographic differences must be considered when evaluating the generalizability of the results, and future qualitative studies should incorporate additional approaches to ensure equitable inclusion or to determine why some groups of participants may be more likely than others to complete open-ended free-response items. Nonetheless, we believe that these findings are meaningful in considering the dynamic relationship between mental health and language use within individuals and over time during the pandemic.

### Mental Health History Impacts Language Use but Not Likelihood of Responding

In contrast to the hypotheses, individuals with a mental health treatment history were not more likely to respond than those without prior histories. However, individuals with mental health treatment histories had more negative sentiment, more FPSP use, higher word count, and used more negative emotional words. When judged according to traditional interpretations of correlation coefficients, correlations between the PPS (a continuous measure of each individual’s likelihood of being a patient based on machine learning) and language features range from negligible to weak at best [75]. However, these associations seem more substantial when judged relative to other relationships between language features and individual differences. One study correlating Linguistic Inquiry and Word Count features and personality traits from Facebook posts highlighted all correlations with a magnitude greater than *r*>0.1 [76]. In addition, 2 meta-analyses of the relationship between FPSP use and depression found a correlation of 0.13 [26] and 0.19 [24]. Tølbøll [24] also reported a correlation of 0.12 between negative emotional word use and depression severity. Edwards and Holtzman [26] commented that the correlation between FPSP use and depression is among the largest when language features are related to individual differences. In this context, the correlation with language features, particularly FPSP use (*r*=0.13), in this study appears to be substantial. Our findings further expand on the literature by using a unique format of text, a sample with nondifferentiated mental health, and a continuous metric of mental health.

### Psychological State Affects Free Response Probability and Sentiment of Responses

Language use was linked not only to mental health history and demographic factors but also to psychological state, as measured by self-reported distress and loneliness. Fluctuations in these factors predict whether an individual is likely to respond at a given time point, the sentiment of the response, and the response length. The fact that psychological state predicted the likelihood of responding suggests that perhaps responding to the free-response question was therapeutic, that is, an outlet to express negative emotions or a method of coping with loneliness. This is supported by the fact that the likelihood of responding and response length decreased over time within individuals, suggesting an overall reduction in motivation to provide free responses over the course of the 6-month study, but that within-subject fluctuations in distress and loneliness all increased response likelihood, response length, and sentiment. Furthermore, this was directly reported by some participants in their free responses, for example, “Thank you for listening”; “I’m disappointed that the study is ending while my world is still so broken. This is one of the few outlets I have to make people listen to how I’m being affected”; “I’m SO bummed this is ending. I’ve enjoyed doing this”; “I’m not happy that the study is ending while my life is still profoundly diminished. This is one of the few places I have a voice to make it known how badly the restrictions and isolation are affecting me”; and “I have appreciated being a part of this research. It helped me to be in better touch with myself.” Of course, other participants felt differently, for example, “I am so glad to finish this off. It was a poorly designed survey (what is ‘normal’?) and it was so biased trying to show the panic over a deadly but unavoidable disease” and “Well well...we are at the end of this study. I hope the NIH will read it’s [*sic*] own articles on the distress caused by wearing masks.”

### Average Sentiment Was Consistently Negative

Across individuals, not surprisingly, the sentiment over the course of the COVID-19 pandemic was generally negative. Other studies performing sentiment analyses on social media with overlapping time ranges during the pandemic have found overall negativity [77,78], overall positivity [73,79,80], or mixed results [81]. This variability is likely a result of the type of language assessed, location of the participants, period, and sentiment analysis algorithm. The consistent negativity we observed could reflect self-selection, that is, individuals may have responded to the question when they felt the worst. This interpretation is supported by results showing that participants were more likely to respond and more likely to have negative responses when they felt more distressed or lonelier. Although the sentiment of this study’s sample cannot be quantitatively compared with others in similar studies, the high prevalence of mental health disorders in this sample (1384/2497, 55.43% of free-response respondents had a history of treatment for mental health) may also have contributed to negativity because of the pandemic’s mental health exacerbations for those with preexisting mental illness [82].

We found that sentiment remained generally stable during the period of most responses and actually increased over time within individuals, contradicting the hypothesis that sentiment would become more negative as quarantine and infection control measures go on, Although increases in global sentiment were not evident until December, around the time of the Food and Drug administration approval of the Pfizer vaccine. The visualization of average sentiment over time suggests that the main source of variability came from major spikes in the TweetEval measure at several points throughout the pandemic, which may have been tied to US national events. For example, negative spikes followed the murder of George Floyd and the Capitol Riots, and positive spikes followed the news of vaccine efficacy and approval. These fluctuations occurred within 2 weeks of the event, which likely reflected the survey being e-mailed to participants every 2 weeks. Importantly, because most responses were between May 2020 and November 2020, interpretations of fluctuations and trends outside of that duration are less reliable. Those inflection points could be true spikes or a result of a few responses. However, these topics also emerged in our TF-IDF analysis, which quantifiably shows a timeline of events during the pandemic based on the unique words used each month. For example, words about the murder of George Floyd appeared in June 2020 and words about the US Capitol attack appeared in January 2021. This suggests that individuals used the free-response item to discuss timely topics of national importance, and the association with changes in sentiment suggests they reported on their feelings about these events.

### Automated and Manual Scoring Provide Insights on Topics Relevant to the Pandemic

An interesting finding is that LDA was successful in identifying relevant topics despite the small size of the data set. Although we used perplexity as a measure to evaluate LDA outputs, the number of topics and topics were human interpretable. We evaluated them using human validations in 3 steps. In addition to the automated sentiment, TF-IDF, and topic model analyses, we used traditional manual coding to evaluate the presence of categories that were expected to be discussed. The most frequent topic across participants was negative impacts on mental health and emotion, including worries, stressors, and concerns (with a frequency of 5159/9741, 52.96%). This is likely to reflect the fact that our study was advertised by the National Institute of Mental Health and had a high (1384/2497, 55.43%) percentage of individuals with a history of mental health treatment. Individuals may have been primed to reflect on the mental health impacts of the pandemic because that was the focus of the overall study, so this result might not be generalizable to the broader population. Participants also frequently discussed social or physical distancing (2475/9741, 25.41%) and policy and government (1938/9741, 19.9%), which included mentions of protests and leadership. These results align with another study [83] that performed a content analysis on social media posts during the pandemic and found that the top negative topic was “Frustration due to life disruptions.” Surprisingly, the number of respondents who spoke about COVID-19–related physical illness or testing and COVID-19–related death remained relatively small. These findings may indicate that what was most salient to many participants at the time of responding was the impact of the pandemic on their mental health and life rather than the infection itself. However, an alternative explanation could be that the number of people who reported contracting COVID-19 was small (of the full survey sample of 3655 participants, only 95 reported testing positive for COVID-19 at any point during the study) or that participants were able to provide all pertinent information using other questionnaire measures, thereby reducing the need to expand on this using the free-response item.

The IRR was calculated for manual coding. Although Fleiss κ and Gwet AC1 statistics were chosen to complement each other, both showed contradicting discrepancies. As expected, Fleiss κ was unusually low for the infrequently annotated categories. Fleiss κ has a notable weakness in dealing with data with low variability [57,58]. In contrast, Gwet AC1 statistic was >0.90 for all but the top 3 annotated categories, suggesting that it artificially inflated the score for categories with low variabilities. This contradiction is best represented by the “Minimal Change to Lifestyle” and “Clarification of survey response” categories, in which Fleiss κ is 0.45 and 0.38 and Gwet AC1 is >0.99 and 0.97, respectively. Similarly, 1 study [84] found large discrepancies between the κ and AC1 statistic with skewed data. In particular, Keener [84] noted that more disagreements between coders led to higher discrepancies between the 2 statistics, with Gwet AC1 statistic being higher, as in this study. Both statistics are reported here to portray IRR in full context.

### Strengths, Limitations, and Outstanding Questions

There were several key strengths to this study. To our knowledge, this is the first study to analyze language during the COVID-19 pandemic for such a long duration, which facilitated the analysis of dynamic within-person changes in mental health and language use. Other studies that applied text mining methods to language during the COVID-19 pandemic had a much shorter duration, captured data from the early stages of the pandemic only, and drew their samples from social media [83,85-90]. In studies on language use in survey responses during the COVID-19 pandemic, this study appears to have the broadest sample and scope. Other studies that analyzed similar texts in surveys during the pandemic targeted more specific populations [74,91,92] or fields [93,94]. We integrated multiple unique and complementary approaches to text analysis, including sentiment analysis, automated text analysis, and human coding, which provided a fuller picture of the qualitative data. Having such a large sample of participants with a history of mental illness provided the power to compare language as a function of whether individuals had prior mental illness. In addition, the literature largely focuses on the relationship between language and depression, whereas our sample represents a broader spectrum of mental health.

Our study also differs from previous work in that we analyzed text from an open-ended free response, whereas most prior studies on mental health and language have been based on data collected from social media. Focusing on texts from social media may bias the findings in favor of respondents who openly post about their mental health on social media. A previous study showed that the same language features that predicted depression and posttraumatic stress disorder on Twitter (assessed through the user’s self-declaration of diagnosis) overlapped with features that predicted demographics and personality [95]. This suggests that the language features most commonly used to predict mental health may be confounded by the language used by those who are more likely to post about their mental health on social media, where most such studies collect their data. Thus, it is important to assess the relationship between language features and mental health in a variety of texts and samples beyond social media and depression. We believe that our findings indicate that even an open-ended free-response measure can provide important insights into general distress and mental health.

There are also limitations to the study that may reduce generalizability or explain why some results did not replicate the literature. First, as mentioned earlier, the sample demographics for the survey were highly skewed, and the participants who self-selected to answer the free-response question were representative of neither the study nor the general population. This problem plagues all studies that analyze free-text comment responses [19]. Second, our study enrolled participants for a 6-month period starting in April 2020, and recruitment ceased in November 2020. We had higher enrollment at the start of the study than at the end; therefore, the response rate was lower after November 2020, reducing the sample size for interpretation after that date. This means that we cannot make reliable inferences on the sentiment or content of a language after the large-scale introduction of vaccines. Third, the variability in response length and frequency might limit the accuracy or generalizability of our findings. Finally, links with mental health relied on self-report measures and only a subset of our participants underwent in-person clinical evaluations. Future studies should compare these approaches in the context of longer free responses and well-characterized clinical samples. Nonetheless, the results replicating the relationships between mental health and various language features found in literature support the validity of this study’s measures.

In conclusion, our analysis of free responses in the context of a study on mental health during the COVID-19 pandemic reveals associations between psychological factors, language, and sentiment and identifies pertinent topics of interest during the first year of the pandemic. We found that mental health history and current psychological state (ie, distress and loneliness) impacted language use, even within a simple open-ended item. In addition, the sentiments of responses varied over time as a function of both within-person dynamics and population-level events. This provides a unique window for mental health during the pandemic, complementing other longitudinal and population-based studies that rely strictly on quantitative measures. Both manual and automated methods were used to characterize the content of these responses, providing insight into the key themes participants raised regarding how the pandemic affected them. However, a skewed sample in various demographics was a limitation and raised questions about what makes one more likely to respond to a survey or a free-text question. Future research should consider adding or using existing free-response questions for quantitative and qualitative language analysis. Future research on language and mental health should also make a deliberate effort to build samples that are diverse in demographics, types of text, and mental health conditions.

## Appendix

Multimedia Appendix 1 Additional information about methods of demographic classification, automated topic analysis and topic modeling, as well as supplemental results (ie, word clouds depicting results of topic modeling) and tables (manual coding categories and definitions; results of separate logistic and linear models).

## Abbreviations

DSM-5: Diagnostic and Statistical Manual-5
FPSP: first-person singular pronoun
IRR: interrater reliability
LDA: latent Dirichlet allocation
NIH: National Institutes of Health
PPS: patient probability score
SVD: singular value decomposition
TF-IDF: term frequency–inverse document frequency
