# Supplementary material for: Trends in Language Use During the COVID-19 Pandemic and Relationship Between Language Use and Mental Health: Text Analysis Based on Free Responses From a Longitudinal Study
Source: JMIR Ment Health. 2023 Mar 1;10:e40899. doi: 10.2196/40899 (PMC9994427; doi:10.2196/40899)
Supplement: Multimedia Appendix 1 [file mental_v10i1e40899_app1.docx]

*Supplementary Materials to accompany*

“Is there anything else you would like to tell us?”: An analysis of language features in text responses to a study on mental health during the COVID-19 pandemic

Rachel Weger^1^, Juan-Antonio Lossio-Ventura^2^, Margaret Rose-McCandlish^1^, Jacob Shaw^2^, Stephen Sinclair^2^, Francisco Pereira^2^, Joyce Chung^2^, Lauren Atlas^1,2,3^

1. National Center for Complementary and Integrative Health, National Institutes of Health, Bethesda, MD
2. National Institute of Mental Health, National Institutes of Health, Bethesda, MD
3. National Institute on Drug Abuse, National Institutes of Health, Baltimore, MD

Correspondence should be addressed to:

Dr. Lauren Y. Atlas, PhD

National Institutes of Health (NCCIH, NIMH, NIDA)

10 Center Drive

Bethesda, MD 20892

301-827-0214

[lauren.atlas@nih.gov](mailto:lauren.atlas@nih.gov)

## Supplemental Methods

###### Demographic classification

Participants provided demographic and clinical history upon enrollment. Participants were allowed to identify their gender using one or more of the following options: male, female, trans male, trans female, genderqueer/gender non-conforming, and/or different identity. Those who selected male and/or trans male were classified as male. Those who selected female and/or trans female were classified as female. Anyone who selected non-conforming and/or different identity was classified as non-binary, regardless of whether they also selected another gender option. All who did not indicate gender as well as one participant who selected male, female, and trans male were classified as unknown gender. In addition, participants were asked to report their income as one of 10 categories. These were aggregated into five categories based roughly on Pew Research Center’s 2020 definitions of lower-, middle-, and upper-class income [1], creating lower- (< $35k), lower/middle- ($35-75k), middle- ($75-100k), upper/middle- ($100-150k), and upper-class (> $150k) categories.

Pre-existing mental health history was determined by whether a participant endorsed history of mental health hospitalization, mental health medication, or drug/alcohol treatment in a clinical history questionnaire collected at baseline. Participants were labeled as having a pre-existing medical illness if they responded that they have or have had cancer, heart disease, high blood pressure, diabetes, stroke, lung disease, liver disease, stomach or intestinal disease, kidney disease, thyroid disease, immune disorder, or other medical disorder.

###### Automated topic analysis

For the TF-IDF and topic modeling analysis only, lemmas related to COVID-19 and (“coronavirus” and “covid19”) and vaccination (“vaccination” and “vaccinate”) were changed to “covid” and “vaccine,” respectively. One of the top lemmas in February and March, “shoot,” was referring to a vaccine “shot” in every use within those two months. However, it was not adjusted since responses from other months contained the lemma “shoot” used in different contexts. The lemma “shot” appears separately in March, a lemmatization of uses of the plural “shots.”

###### Topic modeling

(A) Preprocessing: Responses to the FR question often contain several themes (e.g. a response mentioning both the 2020 U.S. presidential election and COVID-19 vaccines). Thus, to maximize the likelihood of one topic per document, the responses were split into sentences. Next, the text was lemmatized and only nouns, verbs, and adjectives were included. The text was changed to lowercase and stop-words (e.g. “like,” “of,” etc.) and links were omitted.

(B) Topic modeling: We used LDA with Gibbs Sampling for this analysis. Each topic is represented as a mixture of distributions of words. Gibbs Sampling is another technique for parameter estimation and inference of the distributions defined in the LDA model [2]. We implemented this with topicmodels, an R package that provides an interface to the code for fitting LDA models [3]. Parameters of LDA with Gibbs Sampling were set as suggested in previous studies to obtain optimal performance on short texts [4]. The hyper-parameters of LDA were set to α=0.05, the number of iterations were set to 500, and the number of topics (k) were set to 40. Using the 37 topics selected in manual coding as a benchmark, we evaluated k from 25 to 50 topics using perplexity and log-likelihood measures, which evaluate how well a model predicts a sample of new data. 40 was selected as the optimal number for k and the model assigned the sentences to their respective topics.

(C) Multi-word term extraction: we used BioTex, an NLP tool that aims to extract multi-

word terms from text [5]. BioTex implements several measures to extract multi-word terms, such as LIDF-value. LIDF-value stands for "Linguistic patterns, IDF, and C-value information." This ranking measure is a combination of three units: (1) linguistic pattern weights (i.e., the relative frequency), that identify candidate terms based on the most common linguistic patterns (part-of-speech tags also known as lexical categories such as nouns, adjectives, etc.) created from existing Unified Medical Language System (UMLS) metathesaurus terms [6]; (2) IDF (the well-known "Inverse Document Frequency"); and (3) C-value, a measure that seeks to improve the extraction of long terms. C-value was built for extracting multi-word terms [7]. For instance, LIDF-value is able to extract multi-word terms such as "mental health treatment" which is composed of the following lexical categories: “adjective-noun-noun”. Of note, for each topic and its list of sentences, we applied LIDF-value to extract multi-word terms, thus, we applied LIDF-values 40 times (for 40 topics).

Word clouds were created using the Python wordcloud project [8] and compiled the top 5 single-word terms per topic (as determined by LDA in the topicmodels R package) and the top 20 multi-word terms per topic (as determined by LIDF-value)

## Supplemental Results

###### Topic Modeling

All word clouds created through topic modeling are visualized in Supplementary Figure 1.

**Supplementary Figure 1: Topic modeling word clouds**


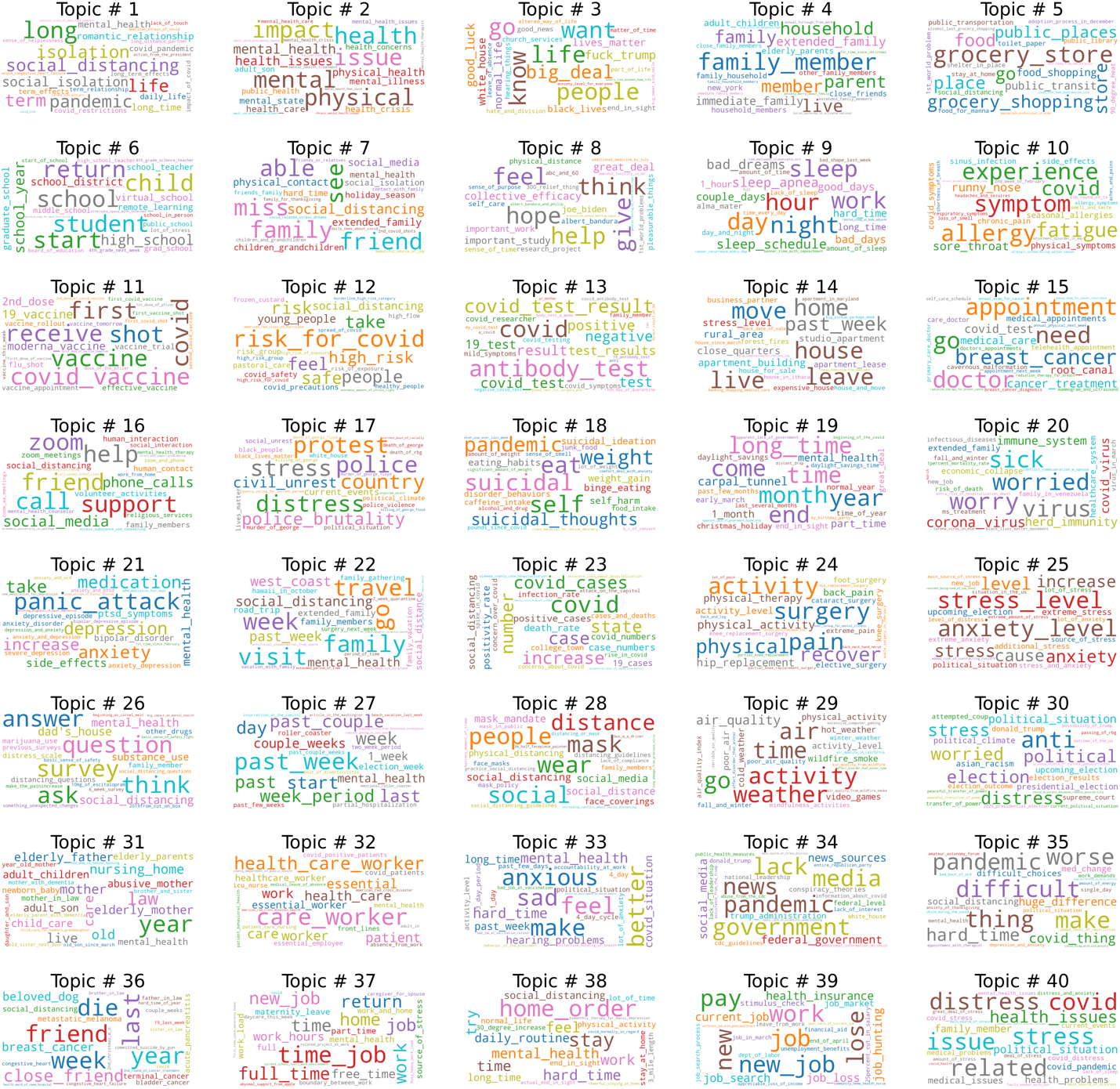


## Supplemental Tables

**Supplementary Table 1: Original manual coding categories and definitions**

| **Revised Category Names** | **Original Category Names** | **Category Definitions** | **Fleiss’ kappa** | **Gwen’s AC1** |
| --- | --- | --- | --- | --- |
| General negative mental health (i.e. Negative emotion or cognitive symptom) | Mental health/emotion | Mentions a negative emotion, like stress, worry, displeasure, sadness, distress, mood, feeling anxious. Mentions cognitive symptoms, such as with memory or focus. Not used for diagnoses of psychiatric illness. | 0.69  (0.68 – 0.71) | 0.70  (0.69 – 0.71) |
| Mention of diagnosis, treatment, suicidality, or domestic violence | Clinically significant information | Specific information about mental/physical illness or treatment in terms of therapy, medication, clinician. Code for anything a clinician should look at, both certain and uncertain diagnoses. Double code with the relevant column for that. diagnosis, in mental health/emotion, or physical health. Domestic violence. | 0.61  (0.59 – 0.63 | 0.88  (0.87 – 0.88) |
| Suicidality | Suicidal thoughts | Any mention of suicidality (ideation, attempt, thoughts, etc). | 0.87  (0.83 – 0.92) | > 0.99  (0.996 – 0.998) |
| Anxiety disorder | Anxiety disorder | Mentions anxiety disorder (OCD, phobia) or panic. | 0.78  (0.74 – 0.82) | 0.99  (0.99 – 0.99) |
| Mood disorder | Mood disorder | Mentions depression or mood disorder. Not just word "depressing". | 0.73  (0.69 – 0.77) | 0.98  (0.98 – 0.98) |
| Other psychiatric diagnosis | Other psychiatric diagnosis | Other psychiatric condition (PTSD, etc.). | 0.50  (0.43 – 0.56) | 0.98  (0.98 – 0.98) |
| Suspected or confirmed COVID illness or test in self | COVID-related illness | Mentions belief about having COVID, COVID test, COVID diagnosis. | 0.86  (0.83 – 0.89) | 0.99  (0.99 – 0.99) |
| Non-COVID-related physical health | Physical health | Mentions non-COVID related physical illness, pre-existing physical condition, current health. | 0.84  (0.82 – 0.85) | 0.95  (0.95 – 0.96) |
| COVID-related risk factors in self | Risk factors | Mentions risk factors such as age. For SELF. | 0.72  (0.65 – 0.78) | > 0.99  (0.991 – 0.995) |
| Pregnancy | Pregnancy | Mentions being pregnant. | 0.78  (0.67 – 0.89) | > 0.99  (0.998 – 0.999) |
| Deferred medical care | Deferred medical care | Mentions deferring care, unrelated to COVID (general). For self or immediate circle. | 0.72  (0.66 – 0.79) | > 0.99  (0.991 – 0.995) |
| Sleep | Sleep | Mentions sleep. | 0.87  (0.85 – 0.90) | > 0.99  (0.989 – 0.993) |
| Change in health behaviors, activities, or hobbies | Change in behaviors | Change in health behavior (eating, exercise - NOT sleep), change in activites, hobbies. Any deviation from previous behavior. | 0.64  (0.61 – 0.68) | 0.95  (0.95 – 0.96) |
| Drugs and alcohol | Drugs and alcohol | Mentions drugs or alcohol (whether increase, decrease, or sober). | 0.83  (0.78 – 0.88) | > 0.99  (0.994 – 0.997) |
| Loneliness/isolation | Loneliness/isolation | Mentions loneliness or feeling isolated. | 0.66  (0.61 – 0.71) | 0.98  (0.98 – 0.99) |
| Experience with social/physical distance and masks | Social/physical distance | Comments about personal experiences with social / physical distance or masks, challenges or life changes due to distancing, personal adherence to social distancing or distancing in one's immediate circle. | 0.49  (0.47 – 0.51) | 0.79  (0.78 – 0.80) |
| Strained relationships | Strained relationships | Interpersonal issues with immediate circle. Mentions needing space or private/quiet time for oneself. | 0.77  (0.74 – 0.80) | 0.98  (0.97 – 0.98) |
| Positive relationships | Positive relationships | Discusses positive relationships with spouse, partner, roommate, family, or immediate circle. | 0.47  (0.39 – 0.55) | 0.99  (0.99 – 0.99) |
| Providing care for dependents | Providing care for dependants | Mentions dependents or care responsibilities, such as child/elder care. Mention of home-schooling. | 0.69  (0.65 – 0.72) | 0.97  (0.97 – 0.97) |
| Health condition or health-related concern about immediate circle | Health of immediate circle | Mentions exposure to COVID, pre-existing conditions, additional risk factors such as age within family or immediate circle. Mention concern about health or health status of member of immediate circle. Can be medical or mental health. | 0.74  (0.72 – 0.76) | 0.92  (0.91 – 0.92) |
| Non-health-related concern for immediate circle | Concern for immediate circle | Mentions concerns about family or immediate circle that are not health-related; general "well-being". | 0.33  (0.28 – 0.38) | 0.96  (0.95 – 0.96) |
| Mention of COVID-related death | COVID death in the family | Mentions a death of confirmed or suspected COVID. | 0.83  (0.76 – 0.89) | > 0.99  (0.996 – 0.998) |
| Mention of non-COVID-related death | Non-COVID related death in the family | Mentions a death unrelated to COVID (at any time). | 0.80  (0.76 – 0.83) | 0.99  (0.98 – 0.99) |
| Experience or concern about reduction/loss in work or unemployment | Reduction in business or work | Mentions loss of income, business, salary, job. Mentions unemployment. Mentions worry or uncertainty about work. | 0.73  (0.70 – 0.77) | 0.98  (0.97 – 0.98) |
| Personal finances | Personal finances | Mentions personal finances, financial stressors. Does not include decreased income due to reduction in business/work. Mentions unemployment benefits. | 0.61  (0.56 – 0.67) | 0.98  (0.98 – 0.98) |
| Other work-related issues | Other work-related issues | Mentions work, work environment/structure, telework. Mention going back to work (also code under Reopening). | 0.69  (0.67 – 0.71) | 0.91  (0.91 – 0.92) |
| School-related changes (student or teacher) | School-related changes | Mentions changes to school, classroom environment/structure, home schooling for self or family. Mentions being a teacher. | 0.78  (0.76 – 0.81) | 0.97  (0.97 – 0.98) |
| Essential worker, or in healthcare | Essential worker, or in healthcare | Is an "essential" worker, or in active social work, public health, mental health, or healthcare practice. SELF | 0.73  (0.69 – 0.77) | 0.98  (0.98 – 0.99) |
| Effects of pandemic on economy, society | Effects on economy, society | Mentions effects on society as a whole or large groups in society, economy, country, world. | 0.44  (0.39 – 0.49) | 0.97  (0.96 – 0.97) |
| Reopening/return to work and interactions with community | Reopening | Mentions reopening or interactions with the community (ex. mask-wearing, others' adherence to social distancing). Mention going back to work or worry about going back to work (also code under Other work-related issues). | 0.57  (0.54 – 0.61) | 0.93  (0.92 – 0.94) |
| Policy/government | Policy/government | Mentions infrastructure (such as general testing), leadership, administration, national and international relations. Reactions to current events about race in America or policing. Mentions protests. | 0.90  (0.89 – 0.92) | 0.96  (0.95 – 0.96) |
| Positive aspects | Positive aspects | Mentions coping strategies, sources of support (marriage, family, socializing, hobbies, etc). Mention positive mental state. Mention "silver linings", such as more free time, more time for exercise, saving money, etc. | 0.66  (0.63 – 0.68) | 0.92  (0.92 – 0.93) |
| Minimal change to lifestyle | Minimal change to lifestyle | Mentions that social distancing measures have not significantly impacted lifestyle (such as that they are introverted, spent a lot of time at home even before, etc.). | 0.45  (0.30 – 0.59) | > 0.99  (0.995 – 0.997) |
| Clarification of survey response | Clarification of survey response | Clarification of survey response. Reference specific question(s) or answer(s). | 0.38  (0.32 – 0.44) | 0.97  (0.97 – 0.97) |
| Survey feedback | Survey feedback | Suggests change to survey or give any other kind of positive or negative feedback. | 0.70  (0.66 – 0.74) | 0.98  (0.97 – 0.98) |
| Other | Other | Anyone who responded with something other than "no", or "N/A", but their response does not fit in previous slots. Only code if the answer has not been coded in any other category. | 0.45  (0.40 – 0.50) | 0.96  (0.96 – 0.97) |
| Vaccine | Vaccine | Any mention of vaccination | 0.96  (0.95 – 0.98) | 0.99  (0.98 – 0.99) |

**Supplementary Table 2: Separate logistic models predicting response likelihood as a function of distress or loneliness.**

|  | *Predictors* | *Odds Ratios* | *CI* | *p* |
| --- | --- | --- | --- | --- |
| Distress model | (Intercept) | 0.36 | 0.34 – 0.39 | **<0.001** |
|  | Week | 0.98 | 0.97 – 0.98 | **<0.001** |
|  | Distress | 1.12 | 1.10 – 1.14 | **<0.001** |
|  | Mean distress | 1.05 | 1.03 – 1.06 | **<0.001** |
|  | Distress * mean distress | 0.99 | 0.98 – 0.99 | **<0.001** |
|  | **Random Effects** |  |  |  |
|  | σ^2^ | 3.29 |  |  |
|  | τ_00_ _SUBJECT_NUMBER_ | 3.08 |  |  |
|  | ICC | 0.48 |  |  |
|  | N _SUBJECT_NUMBER_ | 3163 |  |  |
|  | Observations | 26768 |  |  |
|  | Marginal R^2^ / Conditional R^2^ | 0.017 / 0.492 | |  |
| Loneliness model | (Intercept) | 0.37 | 0.35 – 0.40 | **<0.001** |
|  | Week | 0.98 | 0.97 – 0.98 | **<0.001** |
|  | Loneliness | 1.05 | 1.02 – 1.08 | **0.004** |
|  | Mean loneliness | 1.1 | 1.05 – 1.14 | **<0.001** |
|  | Loneliness * mean loneliness | 0.98 | 0.96 – 1.01 | 0.138 |
|  | **Random Effects** |  |  |  |
|  | σ^2^ | 3.29 |  |  |
|  | τ_00_ _SUBJECT_NUMBER_ | 3.06 |  |  |
|  | ICC | 0.48 |  |  |
|  | N _SUBJECT_NUMBER_ | 3163 |  |  |
|  | Observations | 26334 |  |  |
|  | Marginal R^2^ / Conditional R^2^ | 0.009 / 0.487 | |  |

**Supplementary Table 3: Separate linear models predicting response sentiment as a function of distress or loneliness.**

|  | *Predictors* | *Estimates* | *CI* | *p* |
| --- | --- | --- | --- | --- |
| Distress model | (Intercept) | -0.385 | -0.397 – -0.373 | **<0.001** |
|  | Week | 0.002 | 0.001 – 0.004 | **<0.001** |
|  | Distress | -0.041 | -0.045 – -0.036 | **<0.001** |
|  | Mean distress | -0.02 | -0.023 – -0.017 | **<0.001** |
|  | Distress * mean distress | 0.002 | 0.001 – 0.004 | **<0.001** |
|  | **Random Effects** |  |  |  |
|  | σ^2^ | 0.17 |  |  |
|  | τ_00_ _SUBJECT_NUMBER_ | 0.03 |  |  |
|  | τ_11SUBJECT_NUMBER.KESSLER5_TOTAL.CMC_ | 0 |  |  |
|  | ρ_01_ |  |  |  |
|  | ρ_01_ |  |  |  |
|  | ICC | 0.14 |  |  |
|  | N _SUBJECT_NUMBER_ | 2322 |  |  |
|  | Observations | 9439 |  |  |
|  | Marginal R^2^ / Conditional R^2^ | 0.062 / 0.197 |  |  |
| Loneliness model | (Intercept) | -0.396 | -0.408 – -0.383 | **<0.001** |
|  | Week | 0.003 | 0.002 – 0.004 | **<0.001** |
|  | Loneliness | -0.037 | -0.046 – -0.028 | **<0.001** |
|  | Mean loneliness | -0.028 | -0.034 – -0.021 | **<0.001** |
|  | Loneliness * mean loneliness | -0.001 | -0.008 – 0.005 | 0.726 |
|  | **Random Effects** |  |  |  |
|  | σ^2^ | 0.17 |  |  |
|  | τ_00_ _SUBJECT_NUMBER_ | 0.03 |  |  |
|  | τ_11SUBJECT_NUMBER.UCLA_LONELINESS.CMC_ | 0 |  |  |
|  | ρ_01_ _SUBJECT_NUMBER_ | -0.87 |  |  |
|  | ICC | 0.17 |  |  |
|  | N _SUBJECT_NUMBER_ | 2317 |  |  |
|  | Observations | 9350 |  |  |
|  | Marginal R^2^ / Conditional R^2^ | 0.020 / 0.185 |  |  |

## Supplemental References

1. Parker K, Minkin R, Bennett J. Methodology [Internet]. Pew Research Center’s Social & Demographic Trends Project. 2020 [cited 2021 Dec 20]. Available from: https://www.pewresearch.org/social-trends/2020/09/24/covid-19-financial-hardships-methodology/

2. Wei X, Croft WB. LDA-based document models for ad-hoc retrieval. SIGIR ’06: Proceedings of the 29th annual international ACM SIGIR conference on Research and development in information retrieval [Internet] Seattle, Washington, USA: ACM Press; 2006 [cited 2022 Jan 25]. p. 178–185. [doi: 10.1145/1148170.1148204]

3. Grün B, Hornik K. topicmodels: An R Package for Fitting Topic Models. Journal of Statistical Software 2011;40(13):1–30. [doi: 10.18637/jss.v040.i13]

4. Yan X, Guo J, Lan Y, Cheng X. A biterm topic model for short texts. Proceedings of the 22nd international conference on World Wide Web - WWW ’13 [Internet] Rio de Janeiro, Brazil: ACM Press; 2013 [cited 2022 Jan 25]. p. 1445–1456. [doi: 10.1145/2488388.2488514]

5. Lossio-Ventura JA, Jonquet C, Roche M, Teisseire M. Biomedical term extraction: overview and a new methodology. Inf Retrieval J 2016 Apr;19(1–2):59–99. [doi: 10.1007/s10791-015-9262-2]

6. Bodenreider O. The Unified Medical Language System (UMLS): integrating biomedical terminology. Nucleic Acids Research 2004 Jan 1;32(90001):267D – 270. [doi: 10.1093/nar/gkh061]

7. Frantzi K, Ananiadou S, Mima H. Automatic recognition of multi-word terms:. the C-value/NC-value method. Int J Digit Libr 2000 Aug;3(2):115–130. [doi: 10.1007/s007999900023]

8. Mueller A. WordCloud for Python documentation [Internet]. 2018 [cited 2022 Jun 8]. Available from: https://amueller.github.io/word_cloud/
